# Enhanced Antiproliferative Activity of Docetaxel by Extremely Low Frequency Electromagnetic Fields in MCF-7 Breast Cancer Cells

**DOI:** 10.3390/pharmaceutics17121505

**Published:** 2025-11-21

**Authors:** Maryam Vesal, Yasaman Moazen Safaei, Shahin Ramazi, Abdollah Allahverdi, Parviz Abdolmaleki, Hossein Naderi-Manesh

**Affiliations:** Department of Biophysics, Faculty of Biological Sciences, Tarbiat Modares University, Tehran 14115-111, Iran; mvesal07@gmail.com (M.V.); yasaman.safaie@gmail.com (Y.M.S.); shahinramazi@gmail.com (S.R.); parviz@modares.ac.ir (P.A.); naderman@modares.ac.ir (H.N.-M.)

**Keywords:** breast cancer, docetaxel, frequency electromagnetic field, reactive oxygen species, apoptosis

## Abstract

**Background/Objectives**: Breast cancer remains one of the leading causes of cancer-related mortality in women. While ongoing efforts seek new treatment options, improving the efficacy of existing therapies is equally crucial. Despite advancements in chemotherapy, severe side effects continue to limit treatment success. Combining therapies to enhance efficacy is a promising strategy. Emerging evidence suggests that electromagnetic fields (EMF) can act as chemotherapy enhancers, potentially allowing for lower drug doses. One proposed mechanism is the elevation of reactive oxygen species (ROS), which contributes to cell death. This study explores whether such enhancement exists for chemotherapeutics like docetaxel, where ROS is not the primary, but a contributing, mechanism of action. **Methods**: We investigated the effects of 50 Hz, 20 mT extremely low-frequency EMF (ELF-EMF) exposure to MCF-7 breast cancer cells in the presence of docetaxel. Cytotoxicity was assessed using the MTT assay, while apoptosis, ROS generation, and cell cycle distribution were analyzed via flow cytometry. Morphological changes associated with apoptosis were examined using acridine orange/propidium iodide dual staining and fluorescence microscopy. **Results**: Results showed that 50 μM docetaxel reduced cell viability by 50%, while the combination of ELF-EMF and docetaxel achieved the same effect at a significantly lower docetaxel concentration (14 μM). Flow cytometry revealed similar G2/M phase arrest and apoptotic cell death in the combined treatment group, along with elevated ROS levels. **Conclusions**: These findings suggest that ELF-EMF can potentiate the effects of docetaxel, even when ROS is not the dominant mechanism, supporting its potential as a complementary therapy in breast cancer treatment.

## 1. Introduction

Breast cancer remains a major global health concern due to its high prevalence and mortality rates. According to the World Health Organization (WHO), approximately 2.3 million new cases and 670,000 deaths were reported worldwide in 2022. Although breast cancer primarily affects women, it also occurs in men, accounting for about 1% of all cases. On average, one in twenty women will be diagnosed with breast cancer, and one in sixty will die from the disease. Breast cancer originates in breast tissue and is influenced by multiple risk factors, including genetic predisposition, family history, obesity, alcohol use, reproductive history, tobacco consumption, radiation exposure, and postmenopausal hormone therapy. However, age and female sex remain the most significant risk factors [1].

Breast cancer can be classified into distinct subtypes based on the molecular characteristics of tumor cells [2]. This molecular heterogeneity necessitates a multimodal treatment approach that may include surgery, radiotherapy, endocrine therapy, immunotherapy, and chemotherapy [3]. Ongoing research continues to explore new therapeutic options tailored to these subtypes. Among current strategies, chemotherapy plays a vital role—not only in treating early-stage tumors but also in converting inoperable tumors into candidates for surgical intervention by inhibiting cell proliferation or inducing cell death [3]. However, the non-specific cytotoxic nature of chemotherapeutic agents often affects healthy tissues, particularly within the gastrointestinal, cardiovascular, nervous, immune, and reproductive systems [4]. Consequently, patients commonly experience adverse effects such as hair loss, fatigue, nausea, vomiting, oral mucositis, weight changes, and anxiety [4]. Reducing these side effects while preserving therapeutic efficacy remains a major clinical challenge.

Electromagnetic fields (EMFs) span a wide spectrum of frequencies and are categorized into several types based on their frequency ranges and characteristics. Static magnetic fields occur at 0 Hz; extremely low-frequency EMFs (ELF-EMFs) range from 1 to 300 Hz; low-frequency EMFs extend from 300 Hz to 3 kHz; intermediate-frequency EMFs (IF-EMFs) fall between 3 kHz and 10 MHz; radiofrequency EMFs (RF-EMFs) span from 10 MHz to 300 GHz; and magnetic nanoparticles (MNPs) typically operate in the kHz to MHz range, often involving static or gradient fields. Each EMF type can interact differently with biological systems depending on its frequency, intensity, and duration of exposure [5]. EMFs can enhance chemotherapy efficacy through multiple mechanisms, including increased reactive oxygen species (ROS) generation [6,7], increased drug uptake via altered membrane permeability [8,9,10], disruption of cell cycle progression [11], mitochondrial dysfunction [12], and modulation of apoptosis-related gene expression [6,12], collectively sensitizing cancer cells to treatment. Emerging research suggests a potential link between EMF exposure and ROS generation in cancer cells [13]. While EMF exposure alone may not directly induce cell death, it has been shown to enhance the efficacy of various chemotherapeutic agents [6,7,14,15]. This combinatory effect has been reported with drugs such as doxorubicin [7,14,16], cisplatin [14,15], 5-fluorouracil [15], and methotrexate [15] across different cancer types. Given the potential for enhancement with EMF exposure even in agents where ROS elevation is not the primary mechanism, this study aims to investigate whether taxanes might similarly benefit from such combinatory treatment.

Taxoids are a well-established class of chemotherapeutic agents, with their use dating back to the 1960s. These compounds are derived from the yew tree, a member of the *Taxaceae* family, and share a characteristic taxane core structure. The term “taxoid” broadly refers to both natural and synthetic molecules built around this taxane skeleton. Among the most prominent members of this family is docetaxel (commercially known as *Taxotere*), which was introduced in the 1990s as the second major taxane-based drug after paclitaxel. Docetaxel is a semi-synthetic derivative featuring the classic taxane ring modified with side chains to improve its pharmacological properties. While taxoids are generally lipophilic, docetaxel exhibits improved solubility compared to its predecessor, enhancing its clinical utility [16,17].

Taxoids exert their anticancer effects through multiple molecular pathways. Primarily, they bind with high affinity to β-tubulin, a key component of microtubules, thereby inhibiting microtubule depolymerization [18]. This stabilization disrupts normal cytoskeletal dynamics during mitosis, leading to cell cycle arrest at the G2/M phase and triggering apoptosis [19]. In addition to their effects on the cytoskeleton, taxoids modulate apoptotic signaling by targeting B-cell lymphoma 2 (BCL-2), an anti-apoptotic protein that regulates cell survival. Taxoids downregulate BCL-2 gene expression [20] and promote phosphorylation of the BCL-2 protein [21], thereby impairing its function and facilitating apoptosis [22]. Furthermore, taxoids enhance the expression of tumor suppressors and cell cycle inhibitors such as p21, p27 [23], and p53 [24], contributing to the inhibition of cell proliferation. Another critical mechanism is the induction of ROS, which can surpass the antioxidant capacity of cancer cells and thereby promote oxidative stress [25]. Elevated ROS levels can damage DNA, proteins, and lipids, promote apoptotic pathways, and enhance the cytotoxicity of chemotherapy [26]. Docetaxel, a widely used taxoid, has been shown to disrupt redox homeostasis by reducing glutathione levels, impairing antioxidant enzyme activity, and increasing ROS production and lipid peroxidation [25]. This ROS-mediated imbalance not only hinders cancer cell survival but also highlights the therapeutic potential of targeting redox signaling in combination with conventional chemotherapeutics.

**Scheme 1:**
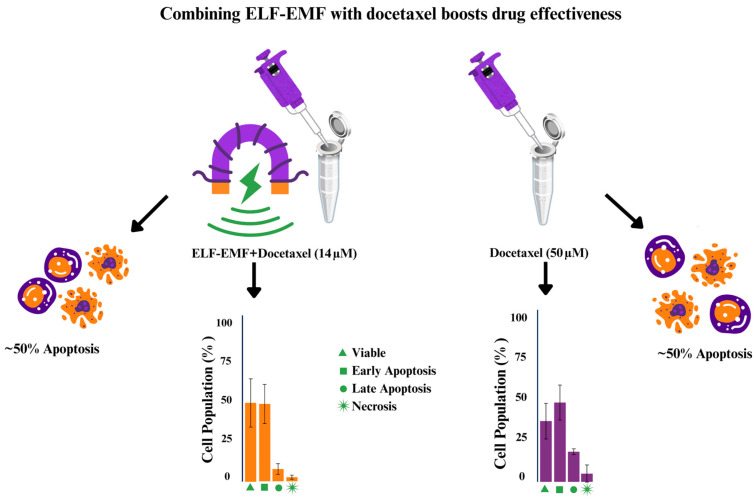
This study evaluated whether extremely low-frequency electromagnetic fields (ELF-EMF) could enhance the efficacy of docetaxel, enabling effective treatment at lower doses. MCF-7 cells were treated under four conditions: control, docetaxel (50 µM), ELF-EMF alone, and a combination of docetaxel (14 µM) + ELF-EMF. The combination of low-dose docetaxel with ELF-EMF produced cytotoxicity comparable to the higher-dose docetaxel alone. The observed combinatory effect appears to be mediated, at least in part, by elevated reactive oxygen species (ROS) levels.

Given the potential of ELF-EMF to enhance chemotherapy, we investigated the combined effects of docetaxel and ELF-EMF on MCF-7 breast cancer cells. As shown in Scheme 1, this approach aims to determine whether ELF-EMF can improve treatment efficacy and potentially reduce side effects.

## 2. Materials and Methods

### 2.1. Materials and Reagents

Docetaxel trihydrate (active pharmaceutical ingredient) was obtained from Actoverco Inc. (Tehran, Iran). A concentrated stock solution was freshly prepared for each experiment by dissolving the compound in dimethyl sulfoxide (DMSO; Sigma-Aldrich, St. Louis, MI, USA, Cat. No. D2438). Before application, the stock solution was diluted in Dulbecco’s Modified Eagle’s Medium (DMEM; Gibco™ 12100061, Carlsbad, CA, USA) to achieve the desired final concentrations. Additional reagents used in this study included: fetal bovine serum (FBS; Bioidea BI-1201, Tehran, Iran), penicillin-streptomycin (Pen-Strep; Bioidea BI-1203), phosphate-buffered saline (PBS; 1X, pH 7.4), and Trypsin-EDTA (0.05%; Bioidea BI-1601) for cell culture maintenance. The following reagents were employed for various assays: propidium iodide (PI; Sigma-Aldrich, P4170), acridine orange (AO; Sigma-Aldrich, A6014), 2′,7′-dichlorodihydrofluorescein diacetate (DCFH-DA; Sigma-Aldrich, D6883) for ROS measurement, Annexin V-FITC apoptosis detection kit (BioLegend, 640906, San Diego, CA, USA), and MTT reagent (Sigma-Aldrich, M2128) for cytotoxicity assays.

### 2.2. Cell Culture

MCF-7 cells (Pasteur Institute, NCBI Code: C135, Paris, France) were cultured in DMEM (Gibco^TM^ 12100061) supplemented with 10% FBS and 1% penicillin-streptomycin (Pen-Strep). For routine passaging, cells were detached using PBS, followed by trypsin-EDTA treatment. MCF-7 cells (Pasteur Institute, NCBI Code: C135) were cultured in DMEM (Gibco^TM^ 12100061) supplemented with 10% FBS and 1% Pen-Strep. For routine passages, cells were detached using PBS followed by Trypsin-EDTA treatment. Cells were incubated at 37 °C with 5% CO_2_ and >95% humidity unless otherwise stated. They were incubated at 37 °C in a humidified atmosphere containing 5% CO_2_, with humidity levels exceeding 95%, unless otherwise specified. Cells were cryopreserved in a freezing medium consisting of 95% complete DMEM and 5% DMSO at −80 °C, then transferred to a liquid nitrogen tank (−196 °C) for long-term storage.

#### 2.2.1. Docetaxel Treatment

Docetaxel trihydrate stock solution (10 mM) was prepared by dissolving 8.65 mg of docetaxel trihydrate in 1 mL of 100 percent DMSO. A fresh stock was prepared for each experimental trial, and any remaining solution was discarded. Intermediate working stocks of 500 µM, 100 µM, 25 µM, and 1 µM were prepared by serial dilution of the 10 mM stock in complete medium. All subsequent dilutions for final treatment concentrations were also made in complete medium. Final concentrations were obtained as follows: 128, 64, 32, and 16 µM were prepared from the 100 µM intermediate stock; 8, 4, and 2 µM were prepared from the 25 µM stock; and 0.5 and 0.25 µM were prepared from the 1 µM stock.

#### 2.2.2. EMF Exposure Setup

The exposure was conducted using an in-house designed magnetic field system, as previously described [7]. It consists of two copper-wire coils (180 turns each) mounted on parallel iron blades (1 m in height and 10 cm^2^ cross-sectional area), connected to an alternating current (AC) power source. These coils can withstand temperatures up to 200 °C. This setup generates a consistent 50 Hz extremely low-frequency electromagnetic field (ELF-EMF) with a fixed intensity of 20 mT under a standard 220 V AC supply. Cell cultures were placed inside a removable plexiglass incubator (23 × 52 × 52 cm) positioned between the iron blades (1 cm apart) and supported on a wooden base for insulation. The incubator was designed to maintain standard cell culture conditions (37 °C, 5% CO_2_, and high humidity) through an automated regulation system equipped with dedicated sensors for temperature, CO_2_, and humidity monitoring. To prevent overheating, the coils were cooled using a remote engine, R12 refrigerant, a condenser, and an evaporator that surrounds the coils. The intensity of the ELF-EMF was measured using a Tesla meter (PHYWE 13610.93, Germany), and the field uniformity was confirmed through simulations performed using CST Studio Suite 2011. To better illustrate the setup, a schematic design has been provided in the supplementary materials (Figure S1).

### 2.3. Acridine Orange/Propidium Iodide (AO/PI) Staining

As an initial qualitative assessment of cell responsiveness to docetaxel under our laboratory conditions, AO/PI dual staining was employed. While acridine orange (AO) can penetrate all cells, propidium iodide (PI) cannot penetrate an intact cell membrane. Therefore, the fluorescence emission spectrum allows differentiation of cell states: green indicates viable cells, bright green to yellow suggests early apoptosis, orange to red is characteristic of late apoptotic changes or necrotic cells.

MCF-7 cells were seeded at a density of 5 × 10^3^ cells/mL in 96-well plates and allowed to adhere for 24 hrs. Subsequently, the cells were treated with docetaxel at concentrations of 25 µM, 50 µM, and 100 µM, alongside a control group. After 24 hrs of incubation, the cells were washed with PBS and stained with a 1:1 mixture of AO and PI (10 µL each, final concentration of 100 µM). Following a 2 min incubation at room temperature, excess dye was removed by washing with PBS. The stained cells were then immediately examined under a fluorescence microscope.

### 2.4. MTT Viability Assay

MCF-7 cells were seeded in 96-well plates at a density of 5 × 10^3^ cells per well in a total volume of 100 μL. The cells were treated with a wide range of docetaxel concentrations (0.25 to 128 µM) for 24 hrs to determine the half-maximal inhibitory concentration (IC_50_). Based on the IC_50_ results, a subsequent experiment was conducted using concentrations ranging from 0.25 to 32 µM in the presence of ELF-EMF.

Following treatment, the culture medium was carefully removed, and 50 μL of MTT solution (0.5 mg/mL in DMEM) was added to each well. Plates were then incubated at 37 °C in a 5% CO_2_ humidified incubator for four hrs to allow the formation of formazan crystals. After incubation, the MTT solution was discarded, and 100 μL of DMSO was added to each well to dissolve the formazan crystals. The plates were gently shaken and incubated in the dark at 37 °C for an additional 30 min. Absorbance was measured at 570 nm using a microplate reader (BioTek Instruments, Winooski, VT, USA).

### 2.5. Annexin v/PI Apoptosis Flow Cytometry

Following the MTT assay, two distinct IC_50_ values were determined: one for docetaxel alone and another for the combined treatment with docetaxel and ELF-EMF. To validate and further characterize the treatment-induced effects, a more precise method was employed: Annexin V/PI staining followed by flow cytometric analysis.

MCF-7 cells were seeded at a density of 2 × 10^5^ cells per well in 6-well plates and divided into four experimental groups: Group I (Control), consisting of untreated cells; Group II (Docetaxel), treated with 50 μM docetaxel; Group III (ELF–EMF), exposed solely to ELF-EMF; and Group IV (Combination), treated with 14 μM docetaxel and exposed to ELF–EMF.

Apoptosis was assessed using the FITC Annexin V Apoptosis Detection Kit. After 24 h of treatment, cells were harvested and washed twice with cold PBS. Each cell pellet was then resuspended in a binding buffer and stained with 3 μL of FITC-conjugated Annexin V and 3 μL of PI 1 mg/mL. Samples were incubated for 20 min at room temperature in the dark.

Stained cells were analyzed using a BD FACSCalibur flow cytometer, and the raw data were processed using FlowJo software (version 10.10.0; FlowJo LLC, Ashland, OR, USA). 

### 2.6. Intracellular ROS Measurement via Flow Cytometry

The primary objective of this study was to investigate the potential combined effect of docetaxel and ELF–EMF, with a particular focus on whether this combination is mediated through increased ROS levels. 

To evaluate intracellular ROS, flow cytometry was performed using 2′,7′-dichlorodihydrofluorescein diacetate (DCFH-DA) as a fluorescent probe. DCFH-DA is a stable, non-fluorescent, and lipophilic compound that easily penetrates cell membranes. Once inside the cell, it is deacetylated by intracellular esterases to form the non-fluorescent 2′,7′-dichlorodihydrofluorescein (DCFH), which is subsequently oxidized by ROS to generate the highly fluorescent 2′,7′-dichlorofluorescein (DCF).

ROS levels were evaluated in MCF-7 cells treated with either docetaxel alone or in combination with ELF–EMF by the fluorescence intensity of DCF. Cells were seeded in 6-well plates at a density of 2 × 10^5^ cells per well and exposed to the IC_50_ concentration of docetaxel, with or without ELF–EMF, for 24 hrs. Following treatment, cells were incubated with 10 μM DCFH-DA in 500 μL of serum-free medium at 37 °C for 45 min. After incubation, cells were washed twice with PBS, trypsinized, collected, and analyzed using a BD FACSCanto II flow cytometer (BD Biosciences, San Jose, CA, USA) with fluorescence detection in the FITC channel. Data analysis was performed using FlowJo software (version 10.10.0; FlowJo LLC, Ashland, OR, USA).

### 2.7. Cell Cycle Analysis

Docetaxel is a known microtubule-stabilizing agent that induces G_2_/M phase arrest. To investigate whether the combination of docetaxel and ELF–EMF produces a similar effect, cell cycle analysis was performed using flow cytometry.

MCF-7 cells were seeded at a density of 2 × 10^5^ cells per well in 6-well plates and divided into four treatment groups. After 24 hrs of exposure to the IC_50_ concentration of docetaxel, either alone or in combination with ELF–EMF, cells were collected for analysis. Following treatment, cells were washed twice with PBS and fixed in 70% cold ethanol at −20 °C overnight to preserve DNA content. Prior to staining, cells were washed again with PBS and resuspended in a staining solution containing 10 μL RNase A (10 mg/mL) and 40 μL propidium iodide (PI, 1 mg/mL). Samples were incubated for 30 min at room temperature in the dark to ensure proper staining.

DNA content was analyzed using a flow cytometer to determine the distribution of cells across different phases of the cell cycle (G_0_/G_1_, S, and G_2_/M). Data were processed using FlowJo software (version 10.10.0; FlowJo LLC, Ashland, OR, USA). 

### 2.8. Statistical Analysis

As mentioned before, ImageJ-FiJi (National Institutes of Health, USA) and FlowJo (version 10.10.0; FlowJo LLC, Ashland, OR, USA) were used to analyze raw data. Statistical significance and graphs were assessed and generated in R (version 4.3.1).

## 3. Results

### 3.1. Dose-Dependent Cell Death in MCF-7 Cells Induced by Docetaxel

**Figure 1:**
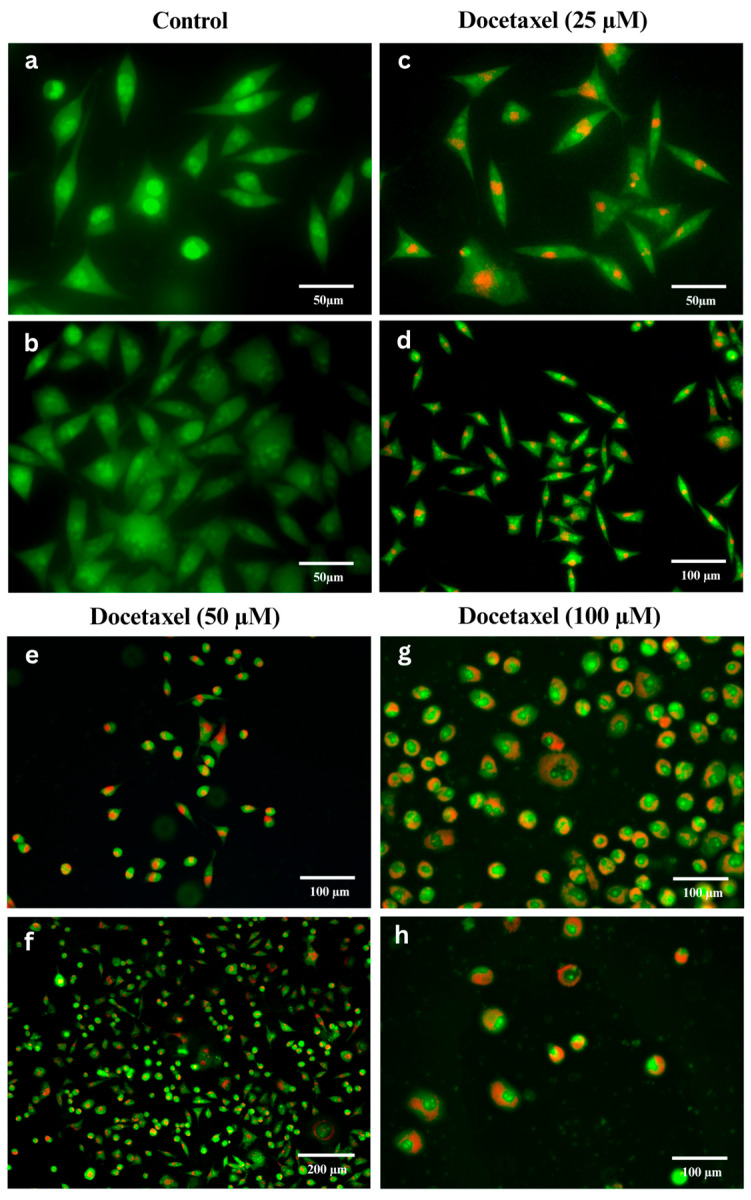
Confirmation of Docetaxel-Induced Cell Death in MCF-7 Cells Using AO/PI Staining. Acridine Orange (AO) stains all nuclei green, while Propidium Iodide (PI) can only enter cells with damaged membranes. As a result, live cells appear green, and yellow-orange fluorescence indicates PI uptake and cell death. (**a**,**b**): Untreated control; (**c**,**d**): Docetaxel (25 µM); (**e**,**f**): Docetaxel (50 µM); (**g**,**h**): Docetaxel (100 µM).

As an initial qualitative assay, acridine orange (AO) and propidium iodide (PI) staining were used to demonstrate morphological changes, dose-dependent effects of docetaxel, and indicators of apoptosis in MCF-7 cells. In Figure 1, cell deformation under docetaxel treatment is clearly visible. The dose-dependent effect is evident from the changing ratio of live to dead cells across different concentrations. In the control group (a, b), nearly all cells remain attached and exhibit a typical polygonal morphology, indicating 100% viability. At 50 µM (Figure 1e,f), approximately half of the cells have lost their normal shape, suggesting partial cell death. At 100 µM (Figure 1g,h), most cells appear amorphous, pointing to widespread cell death. Since PI cannot penetrate live cells, green fluorescence (AO only) indicates living cells. When cells are in apoptotic stages or necrosis, PI enters and emits an orange to red signal. Comparing images (Figure 1c,d), (Figure 1e,f), and (Figure 1g,h), the increasing proportion of orange-stained cells at higher concentrations suggests progressive cell death.

### 3.2. MCF-7 Cell Viability Under Combined Docetaxel and ELF-EMF Treatment

**Figure 2:**
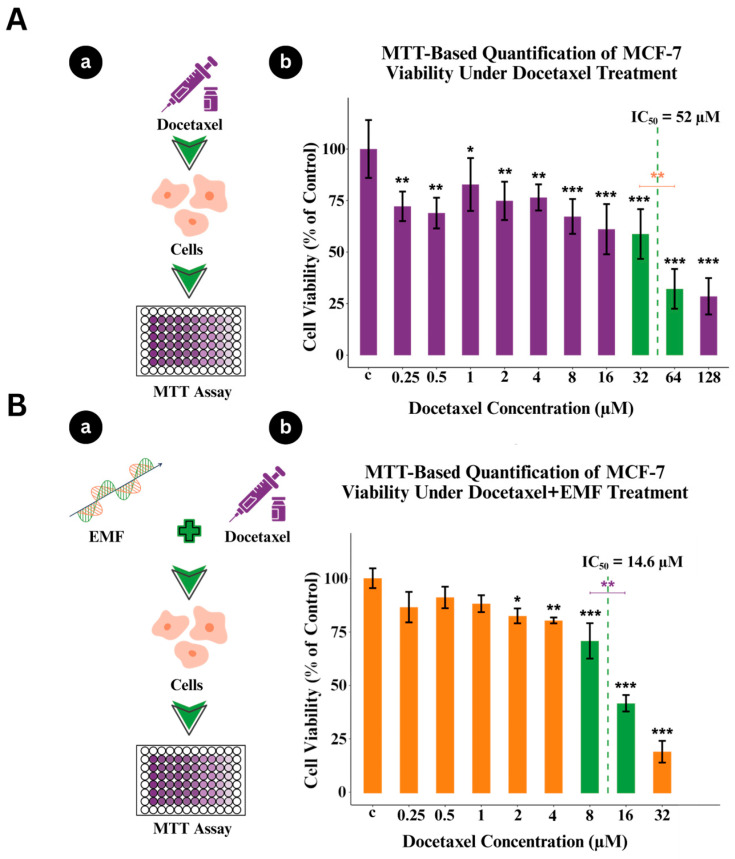
MTT Assay Results for MCF-7 Cell Viability under Docetaxel and Combined Docetaxel + ELF-EMF Treatment. MTT assay was employed to evaluate the viability of MCF-7 cells treated with a range of docetaxel concentrations, both alone and in combination with electromagnetic field exposure. The dashed green line indicates the IC_50_ concentration. The label “c” denotes the control condition. Orange and purple asterisks mark the first statistically significant differences between adjacent concentrations, indicating dose-dependent responses. (**A**) Docetaxel Treatment Only (**a**) Schematic illustration of the experimental setup for docetaxel treatment. (**b**) Quantification of MCF-7 cell viability under varying docetaxel concentrations. (**B**) Docetaxel + ELF-EMF Co-Treatment (**a**) Schematic illustration of the experimental setup for combined docetaxel and ELF-EMF treatment. (**b**) Quantification of MCF-7 cell viability under combined docetaxel and ELF-EMF exposure. Significance levels: *p* < 0.05 (*), *p* < 0.01 (**), *p* < 0.001 (***), and for each concentration *n* = 3.

To examine whether ELF-EMF exposure enhances the therapeutic efficacy of docetaxel in cancer treatment, we performed an MTT assay on MCF-7 cells. The cells were treated with a range of docetaxel concentrations (0.25, 0.5, 1, 2, 4, 8, 16, 32, 64, and 128 µM) either with (Figure 2B) or without (Figure 2A) concurrent ELF-EMF exposure for 24 hrs. Cell viability was assessed by comparing the relative optical density (OD) of treated samples to controls. IC_50_ calculations are reported in Supplementary Figures S2 and S3.

As shown in Figure 2, treatment of docetaxel alone resulted in an IC_50_ of approximately 50 µM. However, when combined with ELF-EMF, the IC_50_ dropped to around 14 µM. This notable reduction indicates that ELF-EMF exposure enhances the efficacy of docetaxel, suggesting a combined interaction. Importantly, ELF-EMF alone did not cause significant cytotoxicity.

Statistical analysis via ANOVA followed by post hoc analysis confirmed statistical significance of these findings, as indicated by the asterisks in the histograms. In the docetaxel-only group, all treated concentrations showed significant differences compared to the control. A clear dose-dependent response was observed beginning between 32 and 64 µM. In the combined docetaxel + ELF-EMF group, significant differences from the control appeared at concentrations of 2 µM and higher. The first notable dose-dependent effect observed between 8 and 16 µM.

### 3.3. Apoptotic Response of MCF-7 Cells Under Combined Docetaxel and ELF-EMF Treatment

**Figure 3:**
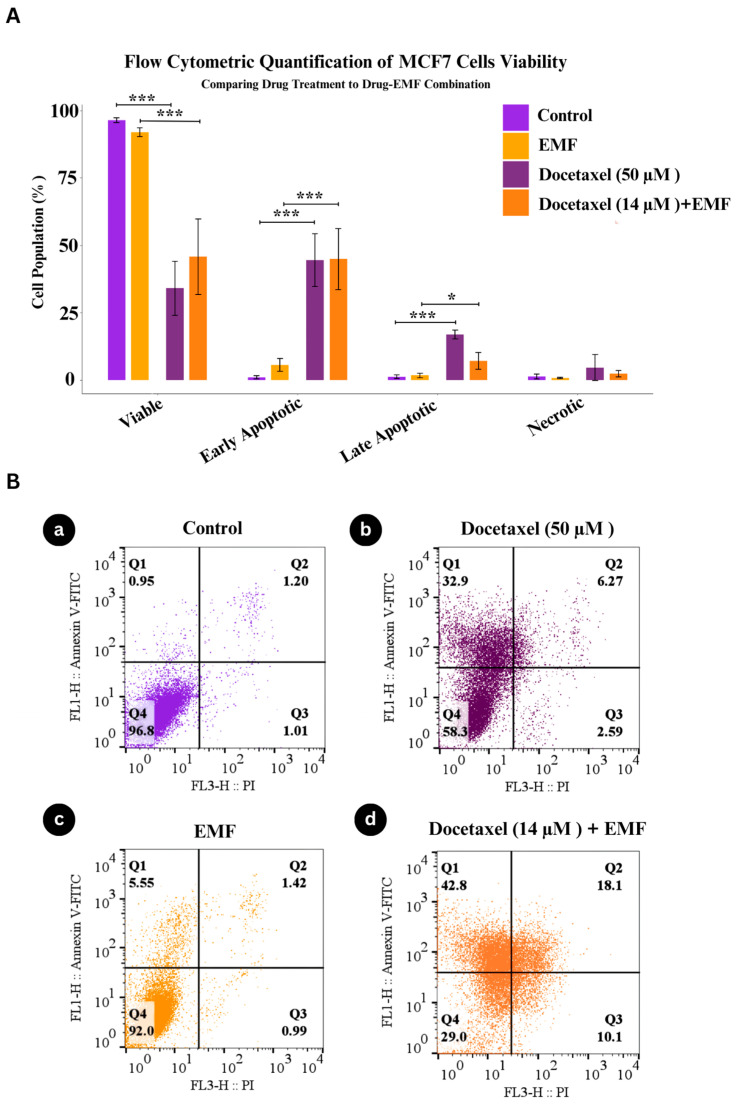
Visualization and Quantification of Apoptotic Status in MCF-7 Cells Treated with Docetaxel and Docetaxel + ELF-EMF. (**A**) Quantitative comparison of apoptotic status in MCF-7 cells following treatment with docetaxel alone or in combination with ELF-EMF. (**B**) Representative Annexin V-FITC/PI dot plots from flow cytometry, showing cell distribution across four quadrants: Q1: early apoptotic, Q2: late apoptotic, Q3: necrotic, Q4: viable. Panels: (**a**)—Control, (**b**)—Docetaxel (50 µM), (**c**)—ELF-EMF only, (**d**)—Docetaxel (14 µM) + ELF-EMF. Statistical significance: * *p* < 0.05, *** *p* < 0.001, and for each concentration *n* = 3.

To compare the apoptotic response of MCF-7 cells under different treatments, we used two IC_50_ concentrations: one for docetaxel alone and one for the combination of docetaxel and ELF-EMF exposure. Previous assays had demonstrated distinct IC_50_ values for each condition. Since the MTT assay provides approximate estimates, flow cytometry was performed here for a more accurate analysis of apoptosis induction. To assess the effects of docetaxel, ELF-EMF, and their combination, cells were stained with Annexin V and PI. This dual staining enables classification of cells into viable, early apoptotic, late apoptotic, and necrotic categories. As shown in Figure 3B, the majority of cells in control (Figure 3B(a)) and ELF-EMF-only (Figure 3B(c)) groups remained viable, indicating that ELF-EMF exposure alone does not induce significant apoptosis.

Interestingly, when docetaxel (14 µM) was combined with ELF-EMF, the proportions of viable and apoptotic cells were similar to those observed in the group treated with 50 µM docetaxel alone. This suggests a potential combined effect of the combined treatment. Statistically, there was no significant difference between the docetaxel (50 µM) and docetaxel (14 µM) and the ELF-EMF groups in the proportions of viable, apoptotic, or necrotic cells. However, in both treatment pairs (control versus docetaxel 50 µM and ELF-EMF -only versus ELF-EMF + docetaxel 14 µM) significant differences were reported in the proportions of viable and apoptotic cells. Exact *p*-values are provided in Table 1.

### 3.4. The Combined Effect of Docetaxel and ELF-EMF Stem from ROS Elevation

**Figure 4:**
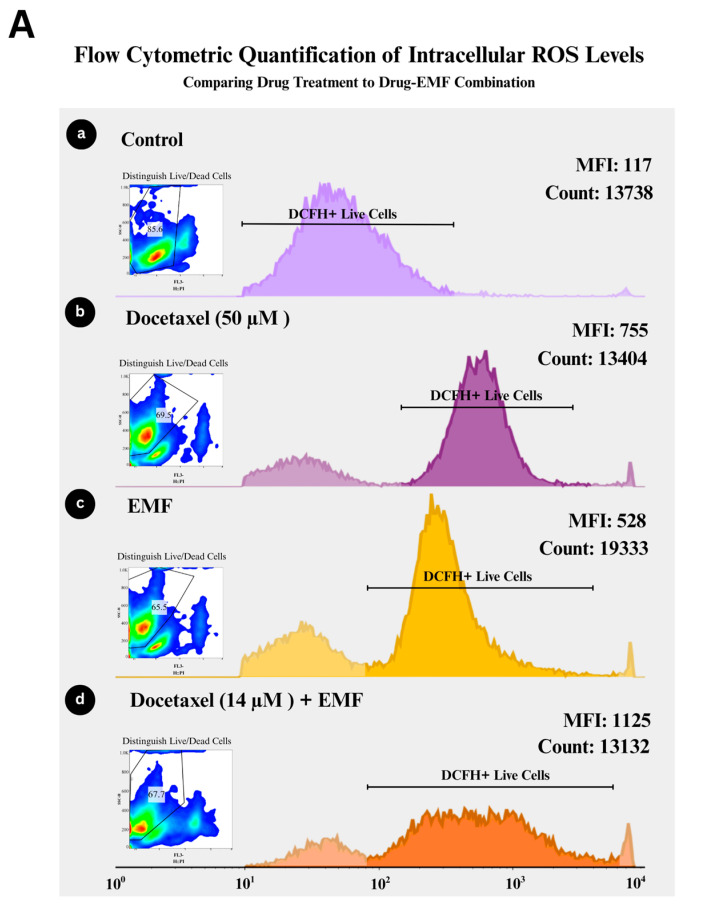

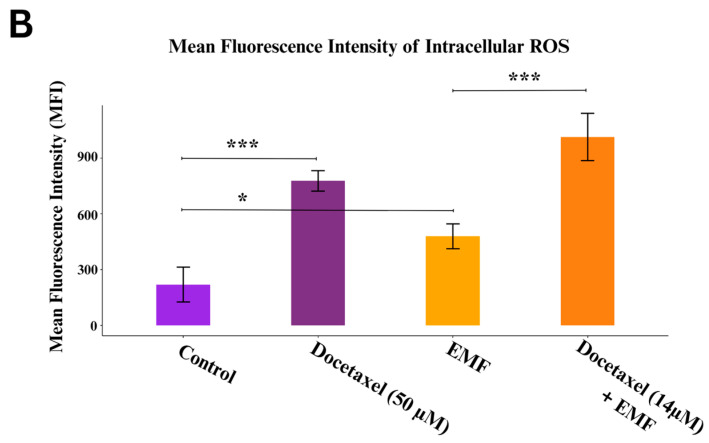
Flowcytometric Quantification of ROS level. (**A**) Representative histograms of ROS levels in different treatment groups. (**a**) Control, (**b**) Docetaxel (50 µM), (**c**) ELF-EMF, and (**d**) Docetaxel (14 µM) + ELF-EMF. The mean fluorescence intensity (MFI) is reported on each graph. Density plots on the left illustrate the gating strategy used to distinguish live cells. In the histograms, darker regions represent DCFH^+^ live cells, while lighter regions correspond to dead cells. (**B**) Quantification of MFI for each treatment group, shown as mean ± SD. Statistical significance is indicated by * *p* < 0.05, and *** *p* < 0.001, and for each concentration *n* = 3.

ROS elevation appears to be a potential shared mechanism contributing to this combined effect. To investigate this, DCFH-DA-stained cells were analyzed using flow cytometry. Mean Fluorescent Intensity (MFI) values were reported, for better comparison. ELF-EMF exposure alone significantly increased ROS levels (Figure 4). Both docetaxel-treated cells (50 µM, Figure 4A(b)) and ELF-EMF-exposed cells (Figure 4A(c)) showed higher ROS compared to control (Figure 4A(a)). However, the combined treatment (docetaxel + ELF-EMF, Figure 4A(d)) produced a much stronger rise. These findings suggest that ROS increase is an important contributor to the observed combinatory effect, even though taxanes like docetaxel primarily act through other apoptotic pathways.

### 3.5. Docetaxel +ELF-EMF Induces G2/M Cell Cycle Arrest

In this study, we evaluated docetaxel and ELF-EMF individually and in combination to assess their potential to enhance therapeutic outcomes. As shown previously, docetaxel induces apoptosis by stabilizing microtubules, leading to G2/M arrest. To compare its cytotoxicity at higher doses (without ELF-EMF) and lower doses (with ELF-EMF), we analyzed cell cycle arrest [21].

**Figure 5:**
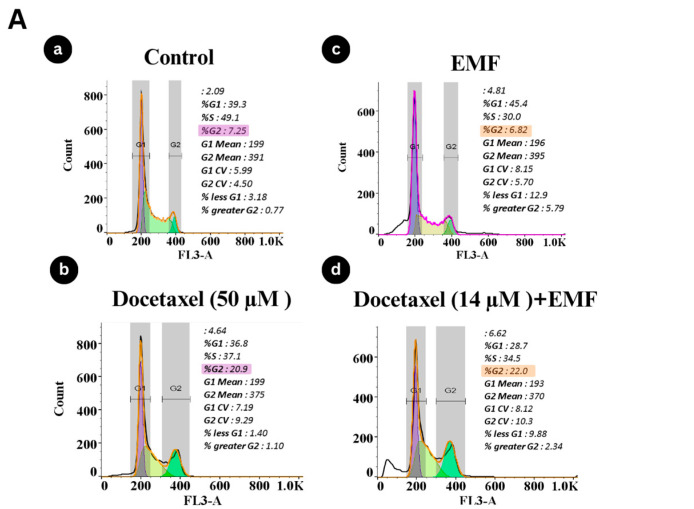

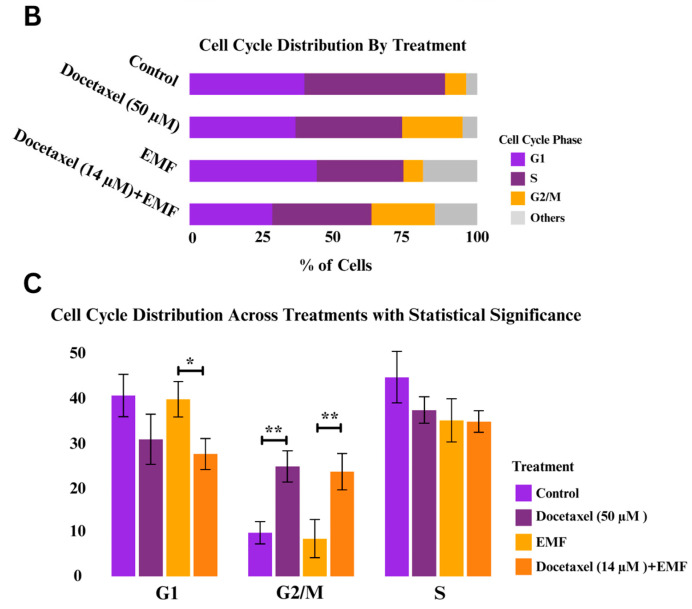
Cell cycle distribution analysis using PI-based flow cytometry. (**A**) Representative histograms show cell cycle distribution under different treatments: (**a**) Control, (**b**) Docetaxel (50 µM), (**c**) ELF-EMF, and (**d**) Docetaxel (14 µM) + ELF-EMF. G2/M percentages are highlighted for each condition. (**B**) Stacked bar plots display the percentage of cells in G1, S, G2/M phases across the four treatment groups: Control, Docetaxel (50 µM), ELF-EMF, and Docetaxel (14 µM) + ELF-EMF. (**C**) Quantitative comparison of cell populations in G1, S, and G2/M phases, with statistical significance indicated (* *p* < 0.05, ** *p* < 0.01), and for each concentration *n* = 3.

Cell cycle distribution was analyzed using PI-based flow cytometry. As shown in Figure 5A,B, both docetaxel (50 µM) and the combined treatment of docetaxel (14 µM) with ELF-EMF induced comparable levels of G2/M arrest relative to their respective control groups (untreated and ELF-EMF alone). Statistical significance for these comparisons is presented in Figure 5C.

## 4. Discussion

Chemotherapy side effects remain one of the most significant challenges in breast cancer treatment. To address this issue, researchers are exploring noninvasive adjunct therapies to improve outcomes while minimizing adverse effects. One promising option is exposure to EMF [27,28], which has demonstrated potential in enhancing the effectiveness of conventional treatments [11].

In recent years, researchers have explored various approaches to applying EMFs in breast cancer models. Based on the timing of exposure, these studies can be categorized into three types: EMF exposure before chemotherapy, continuous exposure during chemotherapy, and pulsed exposure during chemotherapy. For instance, exposing MCF-7 breast cancer cells to ELF-EMFs (50 Hz, 1 mT) prior to treatment with the chemotherapy drug 5-fluorouracil significantly enhanced its antiproliferative effect [29]. Another study showed that concurrent ELF-EMF exposure (50 Hz, 20 mT) and doxorubicin (0.25 µM) exhibited enhanced G_0_/G_1_ cell cycle arrest and increased apoptosis [6]. This study was also conducted on MCF-7 cells for 24 hrs. Additionally, Trebunova et al. examined the combined effect of docetaxel and high-frequency electromagnetic fields (HF-EMF, MHz, 8 W) on MCF-7 cells. Their results suggest that the combined therapy reduced cell viability by 27.5% compared to docetaxel alone [30]. Pulsed EMF (PEMF) exposure has also demonstrated potential: when combined with drugs like etoposide, it increased ROS production, induced DNA damage, and promoted cancer cell death [12]. In a similar study, PEMF was found to enhance the effectiveness of doxorubicin. In this work, three 60 min PEMF sessions per day on MDA-231 cells increased doxorubicin’s antiproliferative effect, while no such improvement was seen with paclitaxel, cisplatin, or 5-fluorouracil. Since paclitaxel is a taxane, this finding suggests that, unlike PEMF, ELF-EMF may have the potential to enhance the effects of taxanes, as seen in our study [31].

Based on previous studies, EMFs are believed to exert combinatory effects with chemotherapeutic agents by promoting mechanisms such as increasing oxidative stress [6,15], affecting membrane permeability [8], improving drug uptake [9], and enhancing DNA damage [32].

As ROS elevation is the most well-established mechanism, these combined effects have primarily been observed with drugs whose cytotoxic activity strongly depends on oxidative stress pathways. Although ROS elevation is not the primary mechanism for some agents, there remains potential for combination with EMF exposure. Therefore, the current study aims to investigate whether taxoids that induce apoptosis through additional mechanisms (while also involving ROS elevation as a secondary effect) can similarly benefit from EMF co-treatment. For this purpose, docetaxel, a microtubule-stabilizing agent from the taxoid family, was selected as the model drug.

To better contextualize our findings, it is important to note that reported IC_50_ values for docetaxel in MCF-7 cells vary widely in the literature. This variability likely reflects differences in experimental design, exposure duration, and the sources of the cell lines or the drug. In our study, we implemented rigorous controls and replicates, relying on consistent in-lab measurements [33]. In line with our results, another study has also reported high IC_50_ values for MCF-7 cell line [34].

Consistent with the apoptotic responses discussed earlier, prior research suggests that exposure to low-frequency, low-intensity EMF does not typically induce apoptosis in MCF-7 cells [6,29]. However, its potential to enhance the effects of chemotherapeutic agents has been repeatedly demonstrated, including with etoposide [12], 5-fluorouracil [29], and doxorubicin [6,35]. Our results indicate a reduction in the IC_50_ value with combined docetaxel + ELF-EMF treatment and compared to docetaxel alone. However, some studies have reported the opposite trend (an increase in IC_50_) although none of these investigations were conducted using docetaxel [36,37].

The observed pattern of cell cycle arrest in our study aligns with findings reported by Vargas and Berchem, who also noted G2/M phase accumulation following docetaxel treatment [38,39]. Although no studies have yet investigated the combined effects of ELF-EMF and docetaxel, previous research demonstrates that lower drug doses combined with ELF-EMF can induce cell cycle arrest comparable to that achieved with higher drug doses alone [6,15,31]. This suggests that the drug is the primary driver of cell cycle arrest, while ELF-EMF may act as a sensitizing agent that enhances the drug’s efficacy. Conversely, a few studies have reported an increase in the S phase following ELF-EMF exposure [40,41]. However, we did not observe this effect in our study, possibly due to differences in research design.

Furthermore, the elevation of ROS appears to be a central mechanism underlying the combination observed in ELF-EMF and chemotherapy combinations. Several independent studies have demonstrated that ELF-EMF can amplify intracellular oxidative stress, thereby enhancing the pro-apoptotic effects of chemotherapeutic agents [6,28,36]. Our results similarly suggest that the enhanced therapeutic effect observed in the ELF-EMF + docetaxel group is at least partly mediated by elevated ROS, reinforcing the notion that ROS serves as a common pathway in this combined interaction.

To address the limitations of this study, we acknowledge that the findings are limited to a single breast cancer cell line (MCF-7). Including additional models, particularly more aggressive phenotypes such as SK-BR-3 or MDA-MB-231, would strengthen the generalizability of our results and help determine whether ELF-EMF exerts similar or distinct effects on cell proliferation and invasiveness across different molecular subtypes. Moreover, our experiments were performed in conventional 2D culture, which does not fully reproduce the complexity of the tumor microenvironment. Future studies employing 3D spheroid or organoid models could provide a more physiologically relevant context for assessing ELF-EMF-mediated modulation of drug sensitivity. Extending exposure duration (e.g., to 48 and 72 hrs) and evaluating a broader range of docetaxel concentrations could also improve the robustness of cytotoxicity assessment. Finally, exploring different ELF-EMF frequencies and intensities, together with gene expression assays targeting oxidative stress, apoptosis, and cell cycle-related pathways, would help clarify the underlying mechanisms driving the observed enhancement of chemotherapy efficacy.

This study shows that combining low-dose docetaxel with ELF-EMF can achieve efficacy comparable to high-dose treatment. This study demonstrates that combining low-dose docetaxel with ELF-EMF achieves efficacy comparable to that of high-dose treatment. However, the therapeutic application of EMF-assisted chemotherapy remains in its early stages. The biological effects of ELF-EMF exposure in humans remain poorly understood, as most studies, including the present work, have been limited to in vitro experiments. Variability in reported outcomes may be attributed to differences in frequency, intensity, and exposure duration. Although low-intensity EMFs are generally considered safe, prolonged or whole-body exposure could potentially lead to unforeseen biological effects [42]. In this study, a low-frequency ELF-EMF was applied at an intensity of approximately 20 mT and a frequency of 50 Hz, representing exposure parameters commonly encountered in urban electricity. This approach aims to minimize potential adverse effects associated with higher-field exposures while enabling evaluation of EMF-assisted chemotherapy in a clinically relevant context. Future in vitro work should expand the dose range, providing a foundation for in vivo studies that confirm these findings and address challenges such as magnetic field penetration and off-target effects.

While ROS amplification may enhance efficacy and allow for lower drug doses with fewer side effects, it also carries the risk of harming nearby healthy tissues. Future studies should therefore determine whether this occurs and develop protective strategies (such as antioxidant co-treatments or targeted delivery) to maximize the therapeutic window. Additionally, well-controlled clinical trials are essential to confirm therapeutic benefits, establish safe exposure parameters, and minimize unintended oxidative stress in healthy tissues.

## 5. Conclusions

Our findings suggest that ELF-EMF could work as a noninvasive partner to chemotherapy drugs. It seems helpful not only for drugs that mainly work by raising ROS levels (like doxorubicin) but also for drugs like docetaxel, which mainly act through other pathways. In the case of docetaxel, ELF-EMF improved its effectiveness, indicating that lower doses might be sufficient, potentially reducing side effects.

## Figures and Tables

**Table 1:** Statistical comparison of viable, apoptotic, and necrotic cell populations across treatment conditions. *p*-values were calculated using one-way ANOVA followed by Tukey’s post hoc test to assess differences between specific treatment pairs. Significant differences were observed in viable and apoptotic populations between control and docetaxel (50 µM), as well as ELF-EMF and docetaxel (14 µM) + ELF-EMF groups. No significant differences were detected in necrosis across conditions. Significant differences (*p* < 0.001) are bold.

Compared Pairs	*p*-Value		
	Live	Apoptotic	Necrosis
Control—Docetaxel (50 µM)	**0.0000984**	**0.0000798**	0.4217
ELF-EMF—Docetaxel (14 µM) +ELF-EMF	**0.00082**	**0.00058**	0.8667
Control—ELF-EMF	0.9171	0.8646	0.9937
Docetaxel (50 µM)—Docetaxel (14 µM) + EMF	−0.4027	0.5066	0.6972

## Data Availability

The raw data supporting the conclusions of this article will be made available by the authors on request.

## Supplementary Materials

The following supporting information can be downloaded at: https://www.mdpi.com/article/10.3390/pharmaceutics17121505/s1, Figure S1: A schematic representation of the ELF/EMF system; Figure S2: Dose–response curve for MCF-7 cells treated with docetaxel; Figure S3: Dose–response curve for MCF-7 cells treated with combined docetaxel and ELF-EMF.

## Abbreviations

The following abbreviations are used in this manuscript:

ELF	Electromagnetic field
ELF-EMF	Extremely Low-Frequency EMF
ROS	Reactive Oxygen Species
BCL-2	B-cell lymphoma 2
AO	Acridine Orange
PI	Propidium Iodide
DCFH-DA	2′,7′-dichlorodihydrofluorescein diacetate
PBS	Phosphate-Buffered Saline
DMSO	Dimethyl sulfoxide
