# Supplementary material for: Enhanced Antiproliferative Activity of Docetaxel by Extremely Low Frequency Electromagnetic Fields in MCF-7 Breast Cancer Cells
Source: Pharmaceutics. 2025 Nov 21;17(12):1505. doi: 10.3390/pharmaceutics17121505 (PMC12735887; doi:10.3390/pharmaceutics17121505)
Supplement: Supplementary file 1 [file pharmaceutics-17-01505-s001.zip › pharmaceutics-3932537-supplementary.pdf]

Supplementary Materials

S1. The ELF-EMF Setup

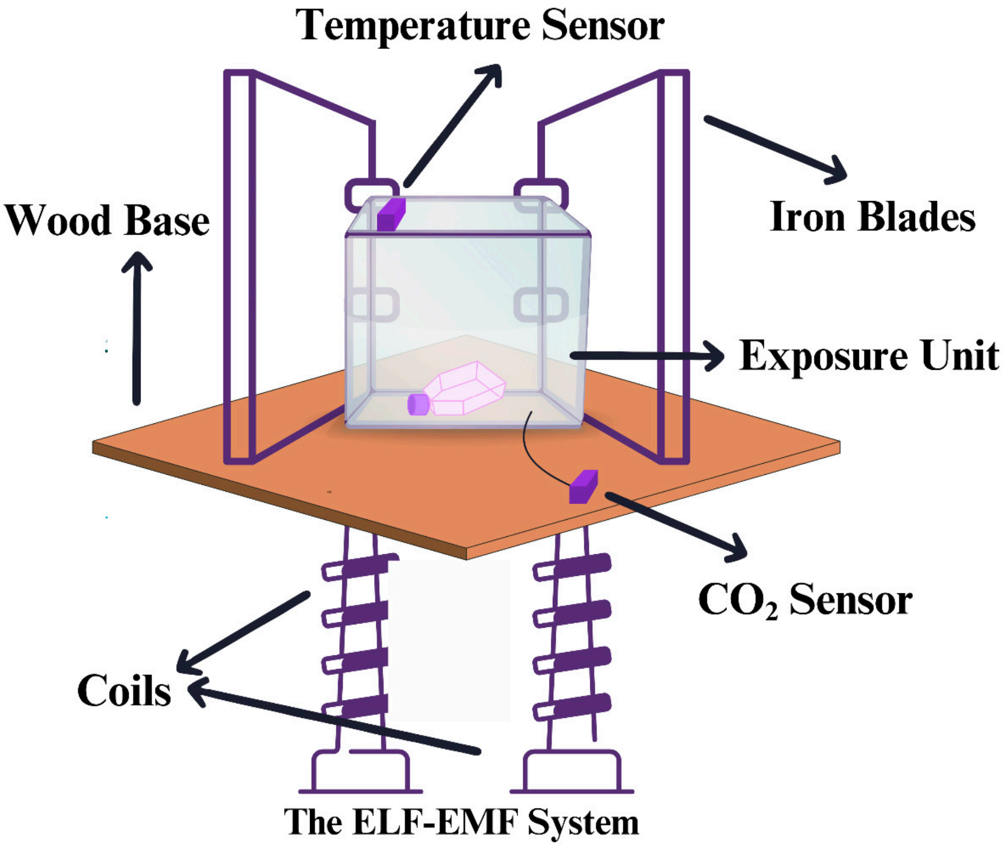

Figure S1. A schematic representation of ELF-EMF system**.** The setup consists of a pair of vertically arranged coils generating the extremely low-frequency electromagnetic field. A wooden base supports the exposure unit, which houses the cell culture plates. Iron blades surround the exposure chamber to help stabilize and shape the generated field. Temperature and CO_2 sensors are positioned inside the exposure unit to monitor environmental conditions throughout experiments.

S2. MTT Dose-Response Curve for MCF-7 cells treated with docetaxel

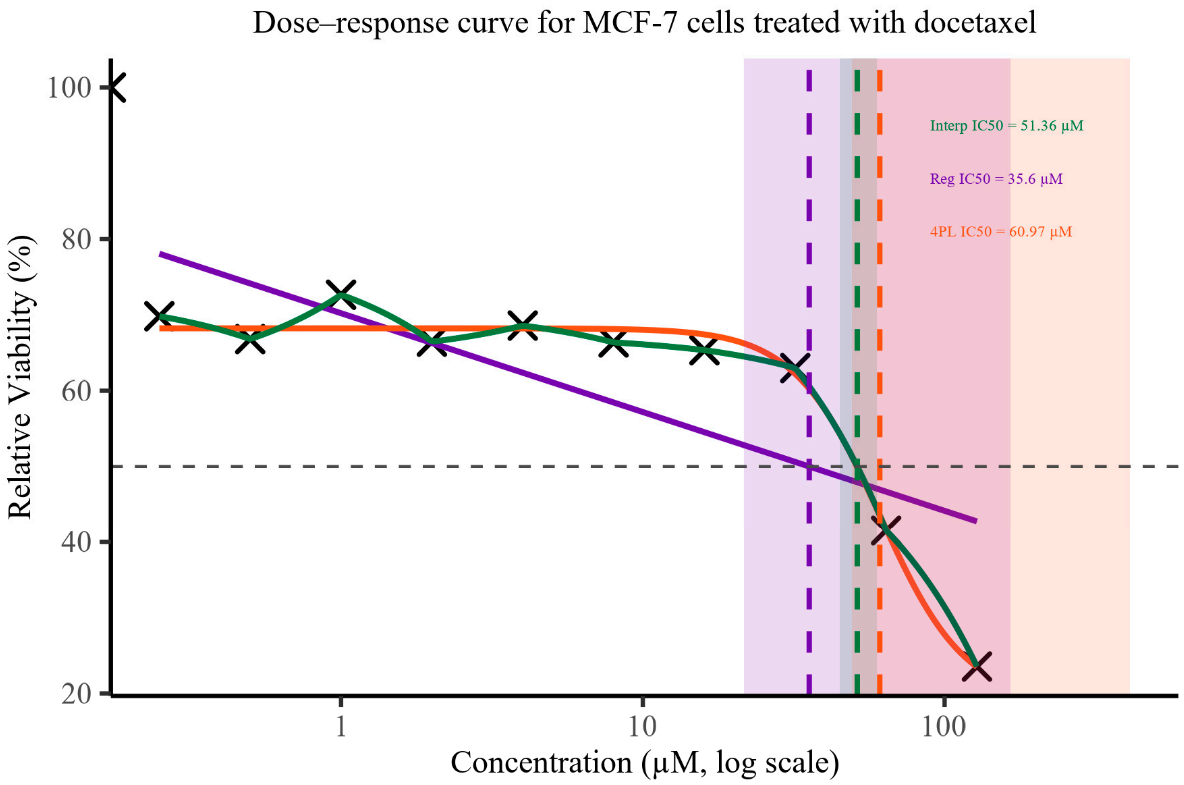

Figure S2. Dose–response curve for MCF-7 cells treated with docetaxel. The plot shows cell viability (%) versus drug concentration (μM, log scale). Data points (cross shapes) represent the mean cell viability at each concentration, normalized to the untreated control. Linear regression on log-transformed concentrations (purple line), 4PL nonlinear model (orange line), and interpolation between measured data points (green line). Vertical dashed lines indicate the IC_{50} values for each model, and shaded areas represent 95% confidence intervals derived from bootstrapping.

MTT assay results for docetaxel treatment were analyzed using three approaches: linear regression on log-transformed concentrations, 4-parameter logistic (4PL) fitting, and simple interpolation between data points. As the plot shows, the regression line deviates strongly from the data points because cytotoxicity curves typically follow a sigmoidal rather than a linear pattern. The 4PL model generally provides the most biologically meaningful IC_{50} by modeling the upper and lower plateaus; however, in this dataset, the lower plateau is not well defined, leading to a wide confidence interval (CI). Interpolation directly estimates the concentration at which viability crosses 50%, yielding a narrower

CI. Based on CI width and visual fit, the interpolation-derived IC_{50} was selected as the most reliable estimate for this dataset.

S3. MTT Dose-Response Curve for MCF-7 Cells Treated with Docetaxel and ELF-EMF

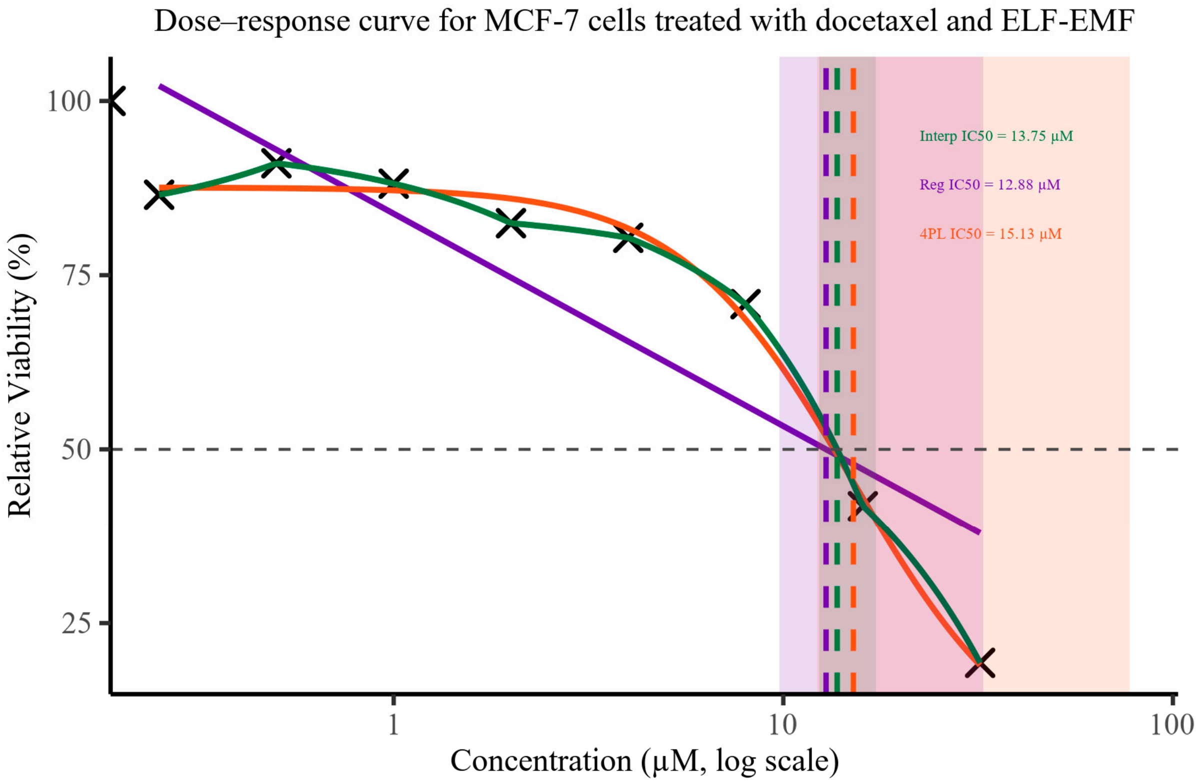

Figure S3. Dose–response curve for MCF-7 cells treated with combined docetaxel and ELF-EMF.

Cell viability was measured using the MTT assay and normalized to untreated controls. Dose–response relationships were modeled using three approaches: linear regression, 4PL nonlinear regression, and linear interpolation with bootstrap confidence intervals. The dashed vertical line indicates the IC_{50}, and the shaded area represents the 95% confidence interval estimated via bootstrapping.

As in the previous analysis, three modeling approaches were applied to the MTT data for combined docetaxel+ELF-EMF treatment: simple linear regression, 4PL nonlinear regression, and linear interpolation with bootstrap confidence intervals. Similar to the docetaxel-only group, the 4PL model provided a smooth nonlinear fit; however, due to incomplete coverage of the lower plateau, CI were calculated to identify the most reliable IC_{50} estimate. Among the models, interpolation with bootstrapping yielded the narrowest CI and best matched the experimental data, suggesting it as the most valid method for IC_{50} determination in this condition.
